# Efficacy of artemether-lumefantrine for treating uncomplicated *Plasmodium falciparum* cases and molecular surveillance of drug resistance genes in Western Myanmar

**DOI:** 10.1186/s12936-020-03376-5

**Published:** 2020-08-27

**Authors:** Yanrui Wu, Myat Thut Soe, Pyae Linn Aung, Luyi Zhao, Weilin Zeng, Lynette Menezes, Zhaoqing Yang, Myat Phone Kyaw, Liwang Cui

**Affiliations:** 1 Department of Cell Biology & Genetics, Kunming Medical University, Kunming, China; 2 Myanmar Health Network Organization, Yangon, Myanmar; 3 Department of Pathogen Biology and Immunology, Kunming Medical University, Kunming, China; 4 Department of Internal Medicine, Morsani College of Medicine, University of South Florida, Tampa, FL 33612 USA

**Keywords:** *Plasmodium Falciparum*, Artemisinin resistance, *pfk13*, *pfcrt*, *pfmdr1*, *pfdhps*, *pfdhfr*, Western Myanmar

## Abstract

**Background:**

Currently, artemisinin-based combination therapy (ACT) is the first-line anti-malarial treatment in malaria-endemic areas. However, resistance in *Plasmodium falciparum* to artemisinin-based combinations emerging in the Greater Mekong Sub-region is a major problem hindering malaria elimination. To continuously monitor the potential spread of ACT-resistant parasites, this study assessed the efficacy of artemether-lumefantrine (AL) for falciparum malaria in western Myanmar.

**Methods:**

Ninety-five patients with malaria symptoms from Paletwa Township, Chin State, Myanmar were screened for *P. falciparum* infections in 2015. After excluding six patients with a parasite density below 100 or over 150,000/µL, 41 *P. falciparum* patients were treated with AL and followed for 28 days. Molecular markers associated with resistance to 4-amino-quinoline drugs (*pfcrt* and *pfmdr1*), antifolate drugs (*pfdhps* and *pfdhfr*) and artemisinin (*pfk13*) were genotyped to determine the prevalence of mutations associated with anti-malarial drug resistance.

**Results:**

For the 41 *P. falciparum* patients (27 children and 14 adults), the 28-day AL therapeutic efficacy was 100%, but five cases (12.2%) were parasite positive on day 3 by microscopy. For the *pfk13* gene, the frequency of NN insert after the position 136 was 100% in the day-3 parasite-positive group as compared to 50.0% in the day-3 parasite-negative group, albeit the difference was not statistically significant (*P *= 0.113). The *pfk13* K189T mutation (10.0%) was found in Myanmar for the first time. The *pfcrt* K76T and A220S mutations were all fixed in the parasite population. In *pfmdr1*, the Y184F mutation was present in 23.3% of the parasite population, and found in both day-3 parasite-positive and -negative parasites. The G968A mutation of *pfmdr1* gene was first reported in Myanmar. Prevalence of all the mutations in *pfdhfr* and *pfdhps* genes assessed was over 70%, with the exception of the *pfdhps* A581G mutation, which was 3.3%.

**Conclusions:**

AL remained highly efficacious in western Myanmar. *Pfk13* mutations associated with artemisinin resistance were not found. The high prevalence of mutations in *pfcrt*, *pfdhfr* and *pfdhps* suggests high-degree resistance to chloroquine and antifolate drugs. The *pfmdr1* N86/184F/D1246 haplotype associated with selection by AL in Africa reached > 20% in this study. The detection of > 10% patients who were day-3 parasite-positive after AL treatment emphasizes the necessity of continuously monitoring ACT efficacy in western Myanmar.

## Background

Malaria remains a major public health problem in tropical and sub-tropical regions of the world. According to the World Malaria Report 2019, it is estimated that there were 228 million malaria cases and 405,000 malaria-related deaths worldwide in 2018 [1]. Currently, malaria control relies primarily on measures targeting vectors (insecticide-treated bed nets and indoor residual spraying) and effective anti-malarial treatment of clinical cases [2]. Since 2001, artemisinin-based combination therapy (ACT) has been recommended as the first-line treatment for *Plasmodium falciparum* [3], and its widespread adoption in malaria treatment policies of endemic nations has played an important role in reducing malaria-related mortality and morbidity. The development of resistance in *P. falciparum* to artemisinins and partner drugs is a major threat to malaria control and elimination [4].

Artemisinin resistance first emerged in western Cambodia in 2007 [5, 6], and has since been detected in all countries of the Greater Mekong Sub-region (GMS), due to spread and/or independent emergence [7, 8]. ACT includes artemisinin or one of its derivatives and a partner drug such as lumefantrine, piperaquine, mefloquine, amodiaquine, and pyronaridine. Evolution of resistance in parasites to the artemisinins and the partner drugs would lead to clinical failures of ACT. In Cambodia, clinical resistance to two ACT, artesunate/mefloquine [9] and dihydroartemisinin/piperaquine (DP) [10–13], has already been identified. To halt the spread of artemisinin resistance in the GMS, ACT efficacy has been monitored in multiple sentinel sites [14–20]. Furthermore, to effectively contain artemisinin resistance in the GMS, countries within the GMS aim to eliminate *P. falciparum* malaria from this region by 2025 [21].

Clinically, artemisinin resistance manifests as delayed parasite clearance with parasite clearance half-life (PC_1/2_) exceeding 5 h, resulting in lingering parasitaemia 3 days after initiation of the treatment [17]. Accurate determination of parasite PC_1/2_ requires sampling of peripheral parasitaemia every 6 h after administration of the artemisinin drug [22]. In resource-limited settings, the day-3 parasite-positive rate can be used as a proxy measure of delayed parasite clearance [23]. Artemisinin resistance affects the ring stage, and dormant ring-stage parasites are able to endure the onslaught of artemisinins and later cause recrudescence of the disease [24]. To capture the ring stage-associated resistance phenotype, an in vitro or ex vivo ring-stage survival assay (RSA) measuring the proportion of the 0–3 h ring-stage parasites surviving 6 h of 700 nM dihydroartemisinin treatment was developed [25, 26]. In 2014, mutations in the propeller domain of the *P. falciparum kelch13* (*pfk13*) gene were identified to be associated with artemisinin resistance [27], providing a molecular marker for surveillance of artemisinin resistance. A large-scale survey of *P. falciparum* populations identified as many as 108 non-synonymous *pfk13* mutations, with wide variation in geographical distribution worldwide; mutations associated with delayed parasite clearance were identified only in Southeast Asia [28]. Likewise, within the GMS, *P. falciparum* populations showed striking disparity in the prevalence and distribution of *pfk13* mutations, with the C580Y and F446I being the predominant *pfk13* mutations in east and west GMS, respectively [27, 29–31]. The NN insertion between amino acids 136 and 137 was associated with artemisinin resistance and its prevalence has increased dramatically over the years along the China-Myanmar border [20, 32].

Molecular markers associated with anti-malarial resistance are useful for resistance surveillance and elucidation of evolution of resistance in parasite populations [33]. Point mutations in the *P. falciparum chloroquine resistance transporter* (*pfcrt*) and the *P. falciparum multidrug resistance* 1 (*pfmdr1*) genes are associated with resistance to chloroquine (CQ) and certain 4-amino-quinoline drugs [34]. In Africa, the extensive deployment of artemether-lumefantrine (AL) has selected parasites with the wild-type N86 and *pfmdr1* haplotype N86/184F/D1246 [35–39]. In the folate biosynthesis pathway, mutations in *P. falciparum dihydrofolate reductase* (*pfdhfr*) and *P. falciparum dihydropteroate synthase* (*pfdhps*) genes as well as amplification of the *GTP*-*cyclohydrolase* gene are associated with resistance to the antifolate drugs sulfadoxine-pyrimethamine (SP) [40, 41].

From 2002, ACT has been deployed for the treatment of falciparum malaria in Myanmar and three ACT, AL, DP and artesunate-mefloquine are recommended [42]. In the GMS, Myanmar has the heaviest malaria burden and its geographical position bridging Southeast Asia and South Asia highlights the need to monitor potential westward spread of resistance. To date, clinical studies to monitor the efficacies of artemisinins or ACT detected artemisinin-resistant *P. falciparum* only in southern and eastern Myanmar [43, 44]. In comparison, ACT remained highly efficacious in northern, northeastern (at the China-Myanmar border) and western Myanmar [19, 45–49]. Molecular surveillance also detected disparate distributions and prevalence of *pfk13* mutations in different regions of Myanmar [29, 30, 46–48, 50], providing a quick assessment of the artemisinin resistance situation. This study evaluated the clinical efficacy of AL for treating falciparum malaria in a western township of Myanmar bordering Bangladesh and India and studied the genetic polymorphisms in genes associated with resistance to AL (*pfk13*, *pfcrt* and *pfmdr1*). Given the extensive use of artesunate-SP in India, this study also genotyped the mutations in the *pfdhfr* and *pfdhps* genes.

## Methods

### Study site and population

**Fig. 1:**
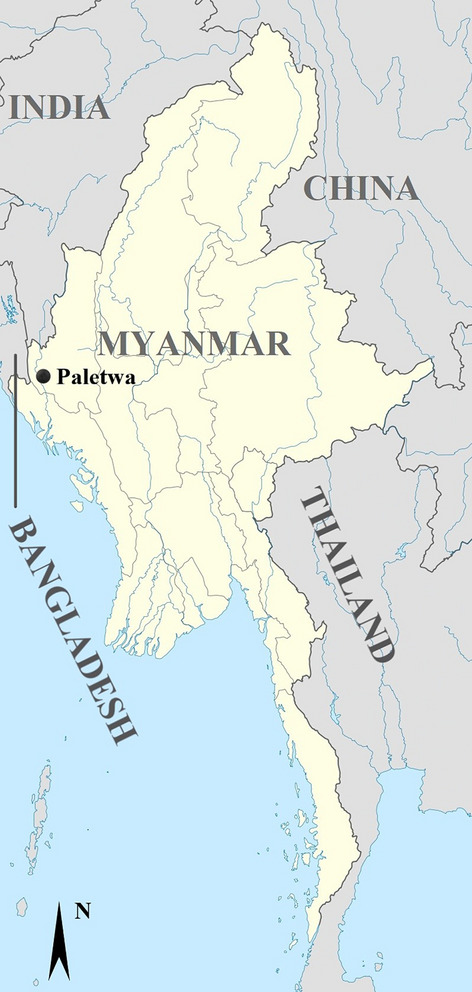
Map of Myanmar showing the study site

Patients presenting with fever (axillary temperature ≥ 37.5 °C) or a history of fever within the previous 24 h and attending clinics at the Paletwa Township, Chin State, Myanmar (Fig. 1) in 2015, were screened for *P. falciparum* infection using the SD Bioline Malaria Ag P.f/Pan (Alere) rapid diagnostic test (RDT). RDT-positive *P. falciparu*m patients were recruited into this study to evaluate the efficacy of AL. Exclusion criteria included severe malaria symptoms, anti-malarial drug use in the previous month, pregnant or lactating women and those with an intention to move out of the study area in the subsequent 2 months. Written informed consent was obtained from the participants or their guardians prior to enrolment. Assent was also obtained from children aged 7 to 17 years. Finger-prick blood samples were collected to make blood smears for microscopic confirmation and determination of parasite density. Patients with parasite density outside the range of 100–150,000 parasites/µL of blood were also excluded. Dried blood spots (DBS) were also prepared on Whatman 3 filter paper, air-dried and stored in individual plastic bags with desiccant. Ethical approval for this study was obtained from the ethical review committee of The Department of Medical Research, Ministry of Health and Sports, Myanmar.

### Treatment and follow-up

RDT-positive *P. falciparum* patients were treated with AL (Coartem^®^) twice daily for a 3-day course. The target dose was calculated according to patient’s body weight (1.3 mg/kg artemether and 8 mg/kg lumefantrine). Patients were instructed to take the tablets and were checked for compliance daily during the follow-up visits on days 1–3. Patients were followed up to day 28 with blood smears collected on days 0, 1, 2, 3, 7, 14, 21, 28, and on any other day if the patient displayed malaria-related symptoms. All collected blood films were assessed for the presence of parasites by microscopy, with genotyping conducted to determine if the parasites were due to a recrudescence or new infection. According to the Myanmar National Malaria Treatment Guidelines, DP is the alternative ACT in case of AL treatment failures.

### *Plasmodium* species identification

Thick smears were stained with 10% Giemsa for 30 min and examined at the field laboratory by microscopy under oil immersion. A smear was considered parasite negative if no parasites were seen after examination of 1000 white blood cells (WBCs). Parasite density, expressed as the number of asexual stage parasites per µL of blood, was calculated by counting the number of asexual stage parasites divided by 400 WBCs, assuming 5000 WBCs/µL blood for patients ≥ 5 years and 7000 WBCs/µL blood for children younger than 5 years [51]. To further confirm *P. falciparum* infections, parasite DNA was extracted from DBS using the QIAamp DNA micro kit (Qiagen, Hilde, Germany). Confirmation of *Plasmodium* infection and differentiation of other *Plasmodium* species including *Plasmodium vivax*, *Plasmodium malariae*, *Plasmodium ovale*, and *Plasmodium knowles*i, were performed using PCR primers and conditions described previously [52, 53].

### Amplification and sequencing of *pfk13, pfcrt, pfmdr1, pfdhps* and *pfdhfr g*enes

The entire *pfk13* gene was amplified using primers and protocol described earlier [30]. Primers for nested PCR of *pfdhps* and *pfmdr1* fragment spanning codons 967–1290 are given in Additional file 1: Table S1. Mutations in exon 2 and 4 of *pfcrt* gene as well as the *pfmdr1* fragment covering codons 77–190 were determined as described previously [54]. The target fragments of *pfdhfr* spanning codons 51–164 were amplified as described earlier [55]. The primary PCR volume was 25 μL, including 1 μM of each primer, 12.5 μL Premix Taq (TaKaRa Biotechnology Co., Ltd. Japan) and 1.5 μL genomic DNA. The nested PCR volume was 50 μL with 2 μM each primer, 25 μL Premix Taq and 2 μL amplified products of the primary PCR. PCR conditions were initial denaturation at 94 °C for 5 min; 35 cycles of 94 °C for 30 s, respective annealing temperatures for 30 s, and 68 °C for 30 s; final extension at 68 °C for 5 min. The amplified PCR products were separated by electrophoresis on 2% agarose gels and visualized after ethidium bromide staining. Then PCR products of different genes were purified and sequenced commercially (Sangon Biotech Co., Ltd. China).

### Sequence analysis

The reference 3D7 sequences for *pfk13* (PF3D7_1343700)*, pfcrt* (PF3D7_0709000)*, pfmdr1* (PF3D7_0523000), *pfdhps* (PF3D7_0810800) and *pfdhfr* (PF3D7_0417200) were obtained from the online database (https://plasmodb.org/plasmo/). All sequences were aligned to respective reference genes by using the DNASTAR (version 7.1) software.

### Statistical analysis

Statistical analysis was carried out using GraphPad Prism 6.0. The general characteristics of samples were described with mean and range. Frequencies of mutations and haplotypes between day-3 parasite-positive and day-3 -negative groups were compared using Fisher’s exact test. *P* value < 0.05 was considered statistically significant.

## Results

### Efficacy of AL in the study population

A total of 95 patients with fever or fever history were screened for *P. falciparu*m infection. Of these 47 were RDT-positive for *P. falciparum* infection and were treated with AL. *Plasmodium falciparum* infections were confirmed by PCR. Microscopic examination of day-0 smears identified 6 samples with parasite density outside the 100–150,000 parasites/µL range, which were excluded from follow-up. The 41 patients included in the efficacy analysis had a median age of 12 years (range 9–60 years), and the majority presented with fever at enrolment (Table 1). Overall, no recurrent cases were detected within the 28 days of the follow-up, giving a 100% adequate clinical and parasitological response. However, there were 5 (12.2%) patients who remained parasite positive on day 3.

**Table 1:** Demographic and clinical characteristics of 41 enrolled patients with *P. falciparum* infection

Number of patients (% male)	41 (51%)
Age in years [median/(range)]	12 (9–60)
Body weight (kg) [mean (range)]	46.3 (29–64)
Patients with fever ≥ 37.5 °C on day 0	33 (80.5%)
Day 0 temperature (℃) [mean (range)]	37.9 (37–39)
Day 0 parasite density/µL [mean (range)]	13631 (131–55,309)

### Mutations in molecular markers of drug resistance

PCR amplification and sequencing were successful from 36 of the 41 patients who were followed for 28 days. There were six samples with double peaks at eight polymorphic sites of the three resistance-associated genes, *pfmdr1*, *pfdhps* and *pfdhfr*, suggesting these samples contained mixed-strain infections (Additional file 2: Figure S1). Sequence data from these samples were excluded from allele and haplotype frequency analysis.

### *Pfk13* gene

For the artemisinin resistance marker *pfk13*, full-length sequences from the 30 samples did not identify any mutations in the propeller domain. The K189T substitution was detected in three samples (10.0%), which were from the day-3 parasite-negative samples. The NN insertion after amino acid 136 was detected in 17 (56.7%) samples (Table 2). The NN insert was present in all day-3 parasite-positive samples compared to 50.0% in day-3 parasite-negative samples, but the difference was not statistically significant (*P *= 0.113).

**Table 2:** Prevalence of mutations in molecular markers of day 0 samples from day 3 positive and negative patients after treatment with artemether-lumefantrine

Gene	Mutation	n (%) of isolates			*P*^a^
		Day 3 positive, n = 4	Day 3 negative, n = 26	Total, n = 30	
*pfk13*	K189T	0 (0.0)	3 (11.5)	3 (10.0)	1.000
	NN insertion	4 (100.0)	13 (50.0)	17 (56.7)	0.113
*pfcrt*	K76T	4 (100.0)	26 (100.0)	30 (100.0)	1.000
	A220S	4 (100.0)	26 (100.0)	30 (100.0)	1.000
*Pfmdr1*	Y184F	1 (25.0)	6 (23.1)	7 (23.3)	1.000
	G968A	0 (0.0)	1 (3.8)	1 (3.3)	1.000
*pfdhps*	S436A	4 (100.0)	20 (76.9)	24 (80.0)	0.557
	A437G	4 (100.0)	26 (100.0)	30 (100.0)	1.000
	K540E	4 (100.0)	23 (88.5)	27 (90.0)	1.000
	A581G	0 (0.0)	1 (3.8)	1 (3.3)	1.000
*pfdhfr*	N51I	4 (100.0)	20 (76.9)	24 (80.0)	0.557
	C59R	4 (100.0)	26 (100.0)	30 (100.0)	1.000
	S108N	4 (100.0)	26 (100.0)	30 (100.0)	1.000
	I164L	3 (75.0)	19 (73.1)	22 (73.3)	1.000

^a^Comparison between the two groups by Fisher’s exact test. Sequences with mixed types were excluded for single mutation analysis

### *Pfcrt* and *pfmdr1* genes

Sequencing data for exon 2 and 4 of the *pfcrt* gene revealed that the mutant K76T and A220S alleles were present in all 30 samples analysed (Table 2). Of the previously reported *pfmdr1* mutations, namely N86Y, Y184F, S1034D, N1042D, and D1246Y, only the Y184F mutation was identified with a prevalence of 23.3% (Table 2). The prevalence of non-synonymous substitution G968A was 3.3% and two synonymous (G182G and T1069T) changes were 16.7 and 3.3%, respectively. There were three *pfmdr1* haplotypes constructed based on the amino acid substitutions (Table 3). Among them, the wild type Y_184_G_968_ had the highest frequency (73.3%), followed by **F**_184_G_968_ (23.3%) and Y_184_**A**_968_ (3.3%).

**Table 3:** Prevalence of pfcrt, pfmdr1, pfdhfr and pfdhps haplotypes in *P. falciparum* isolates from day 3 positive and negative patients after artemether-lumefantrine treatment

Gene	Mutants	Haplotype	n (%)			*P*^a^
			Day 3 positive, n = 4	Day 3 negative, n = 26	Total, n = 30	
*pfk13*	Wild type	K_189_	0 (0.0)	10 (38.5)	10 (33.3)	0.272
	Single	**T**_189_	0 (0.0)	3 (11.5)	3 (10.0)	1.000
		NN K_189_	4 (100.0)	13 (50.0)	17 (56.7)	0.113
*pfcrt*	Double	**T**_76_**S**_220_	4 (100.0)	26 (100.0)	30 (100)	1.000
*pfmdr1*	Wild type	Y_184_G_968_	3 (75.0)	19 (73.1)	22 (73.3)	1.000
	Single	**F**_184_G_968_	1 (25.0)	6 (23.1)	7 (23.3)	1.000
		Y_184_**A**_968_	0 (0.0)	1 (3.8)	1 (3.3)	1.000
*pfdhps*	Single	S_436_**G**_437_K_540_A_581_	0 (0.0)	2 (7.7)	2 (6.7)	1.000
	Double	S_436_**G**_437_**E**_540_A_581_	0 (0.0)	3 (11.5)	3 (10.0)	1.000
		**A**_436_**G**_437_K_540_A_581_	0 (0.0)	1 (3.8)	1 (3.3)	1.000
	Triple	S_436_**G**_437_**E**_540_**G**_581_	0 (0.0)	1 (3.8)	1 (3.3)	1.000
		**A**_436_**G**_437_**E**_540_A_581_	4 (100.0)	19 (73.1)	23 (76.7)	0.548
*pfdhfr*	Double	N_51_**R**_59_**N**_108_I_164_	0 (0.0)	2 (7.7)	2 (6.7)	1.000
	Triple	N_51_**R**_59_**N**_108_**L**_164_	0 (0.0)	4 (15.4)	4 (13.3)	1.000
		**I**_51_**R**_59_**N**_108_I_164_	1 (25.0)	5 (19.2)	6 (20.0)	1.000
	Quadruple	**I**_51_**R**_59_**N**_108_**L**_164_	3 (75.0)	15 (57.7)	18 (60.0)	0.632
*pfdhps*–*pfdhfr*	Quadruple	S_436_**G**_437_K_540_A_581_–N_51_**R**_59_**N**_108_**L**_164_	0 (0.0)	2 (7.7)	2 (6.7)	1.000
		S_436_**G**_437_**E**_540_A_581_–N_51_**R**_59_**N**_108_I_164_	0 (0.0)	1 (3.8)	1 (3.3)	1.000
	Quintuple	S_436_**G**_437_**E**_540_A_581_–**I**_51_**R**_59_**N**_108_I_164_	0 (0.0)	2 (7.7)	2 (6.7)	1.000
	> Quintuple	S_436_**G**_437_**E**_540_**G**_581_–**I**_51_**R**_59_**N**_108_I_164_	0 (0.0)	1 (3.8)	1 (3.3)	1.000
		**A**_436_**G**_437_K_540_A_581_–**I**_51_**R**_59_**N**_108_**L**_164_	0 (0.0)	1 (3.8)	1 (3.3)	1.000
		**A**_436_**G**_437_**E**_540_A_581_–N_51_**R**_59_**N**_108_I_164_	0 (0.0)	1 (3.8)	1 (3.3)	1.000
		**A**_436_**G**_437_**E**_540_A_581_–N_51_**R**_59_**N**_108_**L**_164_	0 (0.0)	2 (7.7)	2 (6.7)	1.000
		**A**_436_**G**_437_**E**_540_A_581_–**I**_51_**R**_59_**N**_108_I_164_	1 (25.0)	2 (7.7)	3 (10.0)	0.360
		**A**_436_**G**_437_**E**_540_A_581_–**I**_51_**R**_59_**N**_108_**L**_164_	3 (75.0)	14 (53.8)	17 (56.7)	0.613

Mutant amino acids are highlighted in bold

^a^Comparison between the two groups by Fisher’s exact test. Sequences with mixed types were excluded for haplotype analysis

### *Pfdhps* and *pfdhfr genes*

There were no wild-type parasites at the *pfdhps* and *pfdhfr* genes (Table 2). Most of the mutations (S436A, A437G and K540E in the *pfdhps* gene; N51I, C59R, S108 N and I164L in the *pfdhfr* gene) exceeded 70%, whereas A581G in *pfdhps* was low at 3.3%. For the *pfdhps* gene, five haplotypes were found in the samples and the triple mutant haplotype **A**_436_**G**_437_**E**_540_A_581_ was the most common (76.7%) compared with the triple mutant haplotype S_436_**G**_437_**E**_540_**G**_581_ (3.3%). The double mutant haplotypes were **A**_436_**G**_437_K_540_A_581_ (3.3%) and S_436_**G**_437_**E**_540_A_581_ (10.0%) and the single mutant haplotype was S_436_**G**_437_K_540_A_581_ (6.7%). For *pfdhfr*, there were four haplotypes and the quadruple mutant haplotype **I**_51_**R**_59_**N**_108_**L**_164_ was found most frequent (60.0%) followed by the two triple mutant haplotypes **I**_51_**R**_59_**N**_108_I_164_ (20.0%) and N_51_**R**_59_**N**_108_**L**_164_ (13.3%) and the double mutant haplotype N_51_**R**_59_**N**_108_I_164_ (6.7%).

Since quintuple mutations in *pfdhps* (437G and 540E) and *pfdhfr* (51I, 59R and 108N) were linked to clinical treatment failure of SP [56], the combination of the *pfdhps* and *pfdhfr* mutations was further evaluated. Of the total of eight *pfdhps*-*pfdhfr* haplotypes, the haplotype with triple *pfdhps* and quadruple *pfdhfr* mutations (**A**_436_**G**_437_**E**_540_A_581_–**I**_51_**R**_59_**N**_108_**L**_164_) was the most common at 56.7%. Three additional haplotypes (**A**_436_**G**_437_**E**_540_A_581_–**I**_51_**R**_59_**N**_108_I_164_, S_436_**G**_437_**E**_540_A_581_–**I**_51_**R**_59_**N**_108_I_164_ and S_436_**G**_437_**E**_540_**G**_581_–**I**_51_**R**_59_**N**_108_I_164_), which all contained the aforementioned quintuple mutations were equally represented at 20.0%.

## Discussion

The emergence and spread of *P. falciparum* resistance to artemisinin in GMS is of great concern and demands the monitoring of clinical efficacy of ACT in malaria-endemic areas of the region. Myanmar occupies an important position in artemisinin resistance containment, because it was among the highest malaria burden countries in the GMS and is geographically linked to the Indian sub-continent [57]. Since the detection of artemisinin resistance in Cambodia [6], delayed parasite clearance in patients after ACT or artesunate treatment was first detected in southern Myanmar in 2010 [42, 44]. One study conducted in northern Myanmar reported 30% of day-3 parasite positivity after treatment with DP in 2013 [48]. The artemisinin resistance phenotype was also documented in eastern (37.1%) [43] and northeastern (23.1%) [20] Myanmar after treatment with artesunate. In southeastern Myanmar, 20% of the cases were still parasitaemic on day 3 after treatment with AL [49]. Despite the presence of artemisinin resistance, ACT still demonstrated high therapeutic efficacies (95.9–100%) in the above areas. In western Myanmar, artemisinin resistance has not been detected. This study confirmed the absence of clinical artemisinin resistance in western Myanmar, with AL demonstrating 100% therapeutic efficacy with no recrudescence within 28 days of follow-up. Although the number of patients tested here was relatively small, the day-28 therapeutic efficacy of AL was consistent with previous studies conducted in the same area [46, 47]. However, the day-3 parasite-positive cases (12.2%) just exceeded the 10% threshold recommended by WHO for suspected emergence of artemisinin resistance.

Artemisinin resistance has been associated with mutations in the propeller domain of *pfk13* [27]. Several mutations including N458Y, Y493H, R539T, I543T, and C580Y have been genetically validated to confer artemisinin resistance [58]. The NN insert outside of the propeller domain has also been reported to be correlated with artemisinin resistance, initially in China-Myanmar border [20]. This insert has increased in prevalence over the years and reached 100% in samples collected in 2014–2016 [32]. No mutations in the propeller domain of the *pfk13* gene were identified in the present study, whereas the NN insert was present in 56.7% patients. This is consistent with a recent study of asymptomatic *P. falciparum* infections in this region showing NN insert as the most popular mutation [59]. Although all of the day-3 parasite-positive samples in the present study harboured NN insert compared to 50% among the day-3 parasite-negative cases, the sample size was too small to perform a robust assessment of the potential association of the NN insert with day-3 parasitaemia. Further investigations are needed to explore the functions of this mutation. The K189T mutation was identified in Myanmar for the first time. This mutation was previously observed in northeast India near Myanmar [60, 61], but it was not associated with increased clearance half-life [14]. The study findings suggest that continuous monitoring of *pfk13* gene mutations and RSA in western Myanmar is warranted.

Several studies investigated the relationship between AL treatment and selection of molecular markers associated with treatment failures. Whereas there was no indication of artemisinin resistance-associated *pfk13* mutations, markedly increased prevalence of *pfmdr1* N86 and *pfcrt* K76 wild-type alleles was associated with extensive use of AL [62]. An in vitro study linked the wild-type *pfmdr1* N86 with reduced lumefantrine activity [36], consistent with the selection of wild-type K76 by lumefantrine [63]. In *pfmdr1*, AL results in the selection of the N86/184F/D1246 haplotype [37–39]. The present study showed that all samples were fixed at K76T and A220S mutations in *pfcrt*, but remained wild type at the *pfmdr1* N86 and D1246. The high prevalence of mutations in *pfcrt* gene may be the result of continued drug pressure of CQ for treating *P. vivax* infections in Myanmar [64]. In *pfmdr1* gene, Y184F had a frequency of 23.3% and there was no statistically significant association between Y184F and the day-3 parasite-positive and -negative phenotypes (*P* = 1.000, Fisher’s exact test). These results were similar to a recent study, which showed the extremely low frequency of N86Y and a moderate prevalence of Y184F in asymptomatic malaria carriers in western Myanmar [59]. The moderate prevalence of the N86/184F/D1246 haplotype associated with AL selection desires further monitoring.

In recognition of the extensive deployment of the artesunate-SP in India, this study also evaluated *pfdhfr* and *pfdhps* mutations and detected high prevalence of *pfdhfr* (N51I, C59R, S108 N and I164L) and *pfdhps* (S436A, A437G and K540E) mutations. Interestingly, these mutations were even more prevalent than previously reported from central Myanmar [59]. The quintuple mutant of *pfdhps* gene (437G and 540E) and *pfdhfr* gene (51I, 59R and 108N) was the significant predictor of clinical treatment failure [56]. Four combined haplotypes containing these quintuple mutations exceeded 70%. In addition, the *pfhdfr* I164L mutation associated with SP failures in Asia [65] also had > 70% prevalence, indicating high-degree SP resistance in this region.

While this study constitutes continued efforts of monitoring the efficacy of anti-malarial drugs in the GMS, it has several limitations. The study reflects the situation that was 5 years ago, and an update is highly desired. The number of patients recruited to this study was small, and an expanded sample size is needed to obtain more accurate estimates of the resistance phenotype. In addition, future studies should extend the follow-up period to 42 days. Furthermore, future studies should also include larger areas along the western Myanmar border to better capture the broad picture of ACT efficacy.

## Conclusions

This study showed that AL was still efficacious for treating uncomplicated falciparum malaria in western Myanmar. Yet, the appearance of day-3 parasitaemia after AL treatment is a warning sign of potential development of artemisinin resistance. Whereas no mutations were identified in *pfk13,* resistance-conferring mutations in *pfcrt*, *pfdhps* and *pfdhfr* genes were highly prevalent, suggesting parasites from this region were resistant to chloroquine and antifolate drugs, and potentially other 4-aminoquinoline drugs. Given the strategic location of Myanmar and the high proportion of *P. falciparum* malaria in western Myanmar, continuous surveillance of therapeutic efficacy of ACT and molecular markers of resistance to both artemisinin and partner drugs, is strongly recommended, which echoes with the WHO’s advice that anti-malarial drug efficacy should be monitored at least once every 24 months in order to provide critical evidence for timely modification of malaria treatment policy [66].

## Supplementary information

**Additional file 1: Table S1.** Primers and annealing temperature of target genes.

**Additional file 2: Fig. S1.** The sequencing chromatograms showing mixed alleles in *pfmdr1, pfdhps* and *pfdhfr* genes of *Plasmodium falciparum* samples from western Myanmar.

## Data Availability

All data and materials are available from the corresponding author.

## Abbreviations

ACT: Artemisinin-based combination therapy
GMS: The Greater Mekong Subregion
AL: Artemether-lumefantrine
DP: Dihydroartemisinin/piperaquine
PC_1/2_: Parasite clearance half-life
RSA: Ring-stage survival assay
*pfcrt*: *P. falciparum chloroquine resistance transporter*
*pfmdr1*: *P. falciparum multidrug resistance 1*
*pfdhfr*: *P. falciparum dihydrofolate reductase*
*pfdhps*: P*. falciparum dihydropteroate synthase*
*pfk13*: *P. falciparum Kelch13*
RDT: Rapid diagnostic test
DBS: Dried blood spots
DNA: Deoxyribonucleic acid
nested-PCR: Nested polymerase chain reaction
