# Supplementary material for: Efficacy of artemether-lumefantrine for treating uncomplicated Plasmodium falciparum cases and molecular surveillance of drug resistance genes in Western Myanmar
Source: Malar J. 2020 Aug 27;19:304. doi: 10.1186/s12936-020-03376-5 (PMC7450958; doi:10.1186/s12936-020-03376-5)
Supplement: Supplementary file 1 — Additional file 1: Table S1. Primers and annealing temperature of target genes. [file 12936_2020_3376_MOESM1_ESM.doc]

| Table S1 Primers and annealing temperature of target genes. | | | | | |
| --- | --- | --- | --- | --- | --- |
| Gene |  |  | Forward primer | Reverse primer | Annealing temperature (°C) |
| *pfk13* | Part1 | nested-1 | 5′ ATTGTTGATGCAAATATTGCTA 3′ | 5′ TGTGCATGAAAATAAATATTAAAGAAG 3′ | 56 |
|  |  | nested-2 | 5′ AAGTGGAAGACATCATGTAACC 3′ | 5′ TGTGCATGAAAATAAATATTAAAGAAG 3′ | 56 |
|  | Part2 | nested-1 | 5′ TATTCGTGTTATAATTTCTCCAAG 3′ | 5′ CAATCGTACTCTTTCCATTTCTA 3′ | 54 |
|  |  | nested-2 | 5′ TATTCGTGTTATAATTTCTCCAAG 3′ | 5′ TCACTAGCATCACTTAATTCCG 3′ | 54 |
|  | Par3 | nested-1 | 5′ TAAAAGCGGAAGTAGTAGCGA 3′ | 5′ TCAACAAGGCTTCACTTTCAC 3′ | 54 |
|  |  | nested-2 | 5′ TCCATCAATTATGAATACCAACA 3′ | 5′ TCAACAAGGCTTCACTTTCAC 3′ | 54 |
| *pfdhps* | SL | nested-1 | 5′ CAGATGGAGGTATTTTTGTTGAA 3′ | 5′ ATCCAATTGTGTGATTTGTCCA 3′ | 56 |
|  | SN | nested-2 | 5′ CAGATGGAGGTATTTTTGTTGAA 3′ | 5′ CCATTGTATGTGGATTTCCTCTT 3′ | 56 |
|  | SD | nested-2 | 5′ TAAATGTGATGCGAAACCAAT 3′ | 5′ ATCCAATTGTGTGATTTGTCCA 3′ | 56 |
| *pfmdr1* | Part4 | nested-1 | 5′ CTACTTATTGCTATTGCTATGTTC 3′ | 5′ AATAGGTACATTTGGTCTTGAA 3′ | 51 |
|  |  | nested-2 | 5′ GTAAGAGCTAGATTAACCAAAAGT 3′ | 5′ AATAGGTACATTTGGTCTTGAA 3′ | 52 |
|  | Part9 | nested-1 | 5′ GAGATGATGGTGGAATAAGAA 3′ | 5′ TTTTATAGATGCAATTCTGTGG 3′ | 52 |
|  |  | nested-2 | 5′ GAGATGATGGTGGAATAAGAA 3′ | 5′ TGGTCCAACATTTGTATCATA 3′ | 51 |
