# Supplementary material for: Efficacy of artemether-lumefantrine for treating uncomplicated Plasmodium falciparum cases and molecular surveillance of drug resistance genes in Western Myanmar
Source: Malar J. 2020 Aug 27;19:304. doi: 10.1186/s12936-020-03376-5 (PMC7450958; doi:10.1186/s12936-020-03376-5)
Supplement: Supplementary file 2 — Additional file 2: Fig. S1. The sequencing chromatograms showing mixed alleles in pfmdr1, pfdhps and pfdhfr genes of Plasmodium falciparum samples from western Myanmar. [file 12936_2020_3376_MOESM2_ESM.doc]

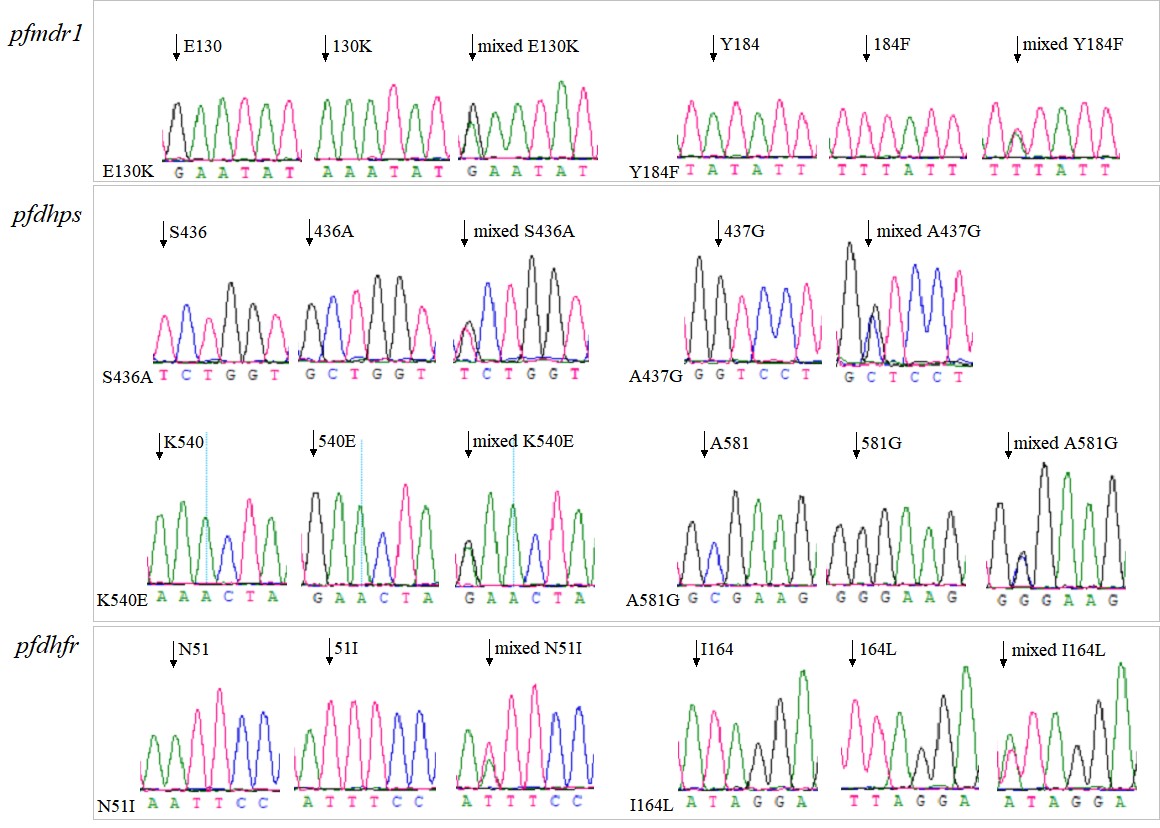


Figure S1 The sequencing chromatograms showing mixed alleles in *pfmdr1, pfdhps* and *pfdhfr* genes of *P. falciparum* samples from western Myanmar.
